# Regression of intestinal failure–associated liver disease by alteration of lipid emulsion regime in home parenteral nutrition: A case report

**DOI:** 10.1002/ncp.70005

**Published:** 2025-07-31

**Authors:** Shahd A. Mohamed, Manoji Gamage, Richard Parker, Rachel M. Brown, Sheldon C. Cooper

**Affiliations:** ^1^ Department of Gastroenterology Queen Elizabeth Hospital Birmingham, University Hospitals Birmingham NHS Foundation Trust Birmingham UK; ^2^ Department of Histopathology Queen Elizabeth Hospital Birmingham, University Hospitals Birmingham NHS Foundation Trust Birmingham UK; ^3^ School of Infection, Inflammation and Immunology University of Birmingham Birmingham UK

**Keywords:** intestinal failure–associated liver disease, lipid injectable emulsions, liver function tests, liver histology, parenteral nutrition

## Abstract

Up to 65% of patients receiving parenteral nutrition (PN) have intestinal failure–associated liver disease (IFALD) and ~25% will have fibrosis on biopsy. First‐generation lipid injectable emulsions (ILEs) are considered a greater risk factor for IFALD compared with newer formulations. We describe a case report of an adult patient with intestinal failure receiving PN who had improvement of liver function tests (LFTs) and regression of fibrosis on liver biopsy following a switch from 100% soybean oil (SO) ILE to SO, medium‐chain triglyceride, olive oil, and fish oil ILE. This supports the only other existing adult case report in the literature to date, which showed histological improvement but no improvements in LFTs.

## INTRODUCTION

Intestinal failure–associated liver disease (IFALD) is an umbrella term used to describe liver injury occurring as a result of several factors related to chronic intestinal failure, including but not limited to parenteral nutrition (PN), and occurring in the absence of another primary parenchymal liver pathology, biliary obstruction, or other hepatotoxic factors.^1^

In adults receiving PN, the reported prevalence of IFALD is up to 65%.^2^ Deranged liver function tests (LFTs) are the most common manifestation of IFALD.^2^ The presence of hepatic fibrosis correlates poorly with biochemical tests,^3^ but approximately a quarter of patients will have fibrosis on biopsy.^2^ The progression to end‐stage liver disease may occur in ~15% of patients.^4^

The risk of IFALD increases with shorter length of bowel,^2^ increased duration of PN,^2^ and greater lipid intake,^2^ and infectious episodes account for the majority of acutely deranged LFTs. First‐generation lipid injectable emulsions (ILEs) are also considered a greater risk factor for IFALD than newer formulations.

## CASE HISTORY

A 21‐year‐old previously healthy man presented in 2001 with a spontaneous small bowel volvulus leading to superior mesenteric artery occlusion and small bowel infarction. Postoperatively, he had 40 cm of small bowel remaining in continuity with his colon, requiring PN 7 nights per week; initially all containing 100% soybean oil (SO) ILE.

**Figure 1:**
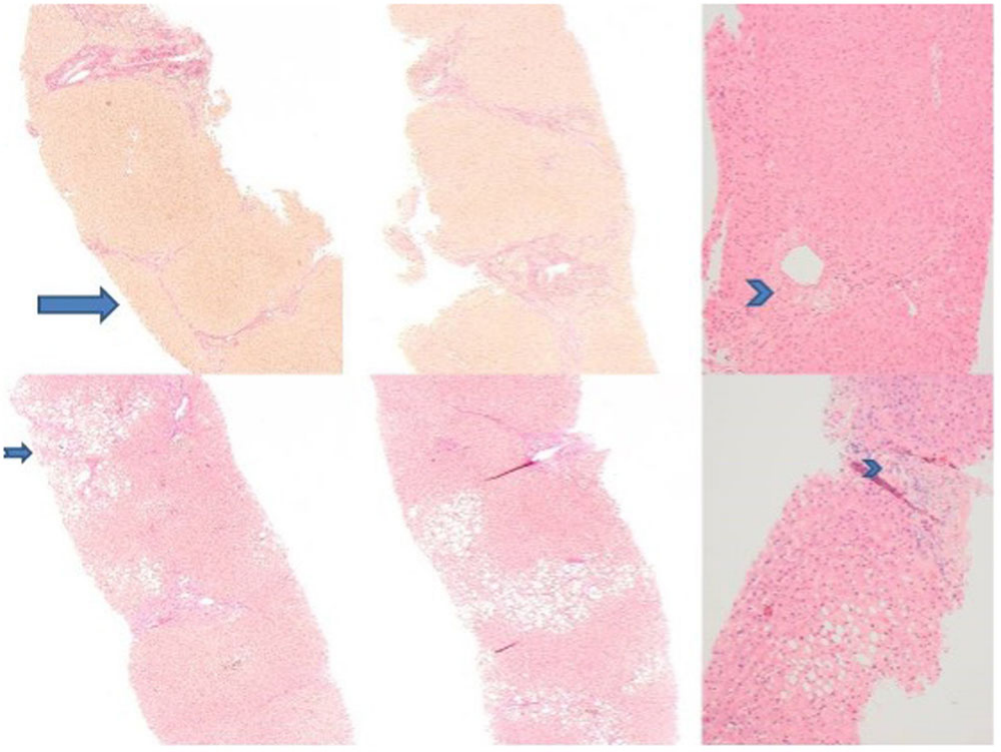
Liver histology. Serial liver histology sections illustrating changes between 2011 (top panel) and 2015 (bottom panel). Top left: demonstrating bridging fibrosis. Hematoxylin–Van Gieson (HVG) stain original magnification ×40. Top right: Hematoxylin and eosin (H&E) stain ×100 showing bile duct paucity. The chevron marks unaccompanied artery. Bottom panel: 2015 biopsy. Less fibrosis is demonstrated, no bridging fibrosis seen. Steatosis has increased during this time period, and there is some pericellular fibrosis in a perivenular region (notched arrow). Bottom right: Chevron again marks an unaccompanied artery.

In 2001, routine liver biopsies showed minor cholestatic changes. In 2002, repeat biopsies showed cholestasis and mild fibrosis. In 2004, the proportion of bags containing SO ILE were reduced to 3 out of 5 nights receiving PN (Table 1). By 2008, this reduction had resulted in weight loss, resulting in an increase in SO ILE to five nights, providing 56 g of ILE per night. His liver tests remained abnormal in 2009, so a liver biopsy was conducted, which showed mild bridging fibrosis. Subsequently, the lipid source was changed from five nights of SO ILE to two nights of SO, medium‐chain triglyceride, olive oil, fish oil (SO‐MCT‐OO‐FO) ILE, which provided 101 g of ILE per night. SO‐MCT‐OO‐FO ILE contains 30% SO, 30% MCT, 25% OO, and 15% FO. Despite this, his liver tests remained abnormal in 2011, and this prompted a further biopsy in May 2011 (Figure 1) in which moderate bridging fibrosis and bile duct paucity was observed with minimal fat. Consequently, the patient was referred for review by a small intestinal and multivisceral transplantation team should progression occur but was not listed for transplantation.

**Table 1:** Summary of liver biochemistry, weight, and PN prescription.

	2001	2002	2004	2005	2007	2008	2009	2010		2011	2012	2013	2014	2015
HPN prescription														
No. of days per week	7	7	5	5	5	5	5	5		5	5	5	5	5
No. of nights of lipid	7	7	3	3	3	5	2	2		2	2	2	2	2
No. of nights of glucose	0	0	2	2	2	0	3	3		3	3	3	3	3
Lipid type	SO ILE	SO ILE	SO ILE	SO ILE	SO ILE	SO ILE	SO‐MCT‐ OO‐FO ILE	SO‐MCT‐ OO‐FO ILE		SO‐MCT‐ OO‐FO ILE	SO‐MCT‐ OO‐FO ILE	SO‐MCT‐ OO‐FO ILE	SO‐MCT‐ OO‐FO ILE	SO‐MCT‐ OO‐FO ILE
Nonnitrogen calories (kcal)	2011	Unavailable	2111	2111	2000	1961	2511	2511		2511	2861	2861	2861	2871
Lipid constituent (g/day)	101	Unavailable	101	101	100	56	101	101		101	115	115	115	116
Glucose constituent (g/day)	250	Unavailable	250	250	250	350	375	375		375	425	425	425	427
Glucose bag kcal/glucose constituent (g/day)	0	Unavailable	1600/400	1600/400	1600/400	0	2500/625	2500/625		2500/650	2600/650	2600/650	2600/650	2610/652
Nutrition status														
Weight (kg)	66.9	63.3	63.4	67	63.5	62.3	60.0	64.5		65.8	65	70.3	69.2	67
BMI	19.3	18.2	18.3	19.4	18.4	18	17.3	19		19	18.8	20.3	20	19.4
Biochemistry														
Bil (μmol/L)	62	70	Unavailable	54	55	33	19	29		17	13	30	18	22
ALP (IU/L)	196	144	Unavailable	468	429	345	445	412		370	315	231	228	258
ALT (IU/L)	N/A	518	Unavailable	302	161	92	177	106		151	85	133	128	102
Albumin (g/L)	33	44	Unavailable	51	47	28	43	48		46	13	45	44	36
Liver biopsy	x	x					x			x				x
Liver biopsy result	Minor cholestatic changes.	Cholestasis and mild fibrosis.					Mild bridging fibrosis.			Progressive portal changes with moderate fibrosis. Bile duct paucity noted.				No bridging fibrosis. Increased steatosis.

Abbreviations: ALP, alkaline phosphatase; ALT, alanine aminotransferase; BMI, body mass index; FO, fish oil; HPN, home parenteral nutrition; ILE, lipid injectable emulsion; MCT, medium‐chain triglyceride; OO, olive oil; PN, parenteral nutrition; SO ILE, soybean‐oil.

In January 2015, his LFTs had shown some improvement but remained raised: alkaline phosphatase 258 IU/L, alanine aminotransferase 102 IU/L, and bilirubin 22 μmol/L. Further liver biopsy in 2015, to assess disease progression and response to the PN ILE alteration, demonstrated reduced fibrosis compared to previous biopsies and bridging fibrosis was no longer present (Figure 1). Steatosis had increased and there was some pericellular fibrosis in a perivenular region. His LFTs had remained stable in the subsequent 8 years and his body mass index was restored to 20 from 19.3 (kg/m^2^). He had never had any catheter‐related bloodstream infection (CRBSI) events, which may confound his liver histology or LFTs, and his hemolysis screen result was negative.

## ETHICAL APPROVAL STATEMENT

The patient has provided written consent for anonymized publication of his case report. Ethical approval was not required for publication of this case.

## DISCUSSION

Deranged liver tests are present in 19%–95% of patients with chronic intestinal failure^5^ and can be caused by sepsis,^6^ lack of enteral nutrition,^7^ and short bowel length.^8^ In this current case, the patient never had a CRBSI, gallstones, history of alcohol excess, or viral hepatitis. Distinguishing whether a patient has IFALD is challenging when there is no consensus for a unifying definition^9^ or diagnostic criteria.^10^ Selecting patients who require a liver biopsy remains a dilemma given that liver blood tests poorly correlate with the degree of liver fibrosis on histology in patients receiving PN.^11^

Apart from LFTs, there is no guidance on noninvasive methods for monitoring progression of liver disease in patients with intestinal failure (IF), which could prompt a liver biopsy. Noninvasive methods to detect fibrosis based on certain biochemical panels include the Fibrosis‐4 score (FIB‐4). FIB‐4 uses a formula derived from a patient's age, alanine transaminase, aspartate transaminase, and platelet count.^12^ It is used in patients older than 35 years with known risk factors for liver disease (eg, chronic hepatitis B and C viruses, metabolic dysfunction–associated fatty liver disease, alcohol‐related liver disease) to prompt further testing for those with indeterminate results or positive results (unless there is evidence of clinical cirrhosis).^13^ FIB‐4 has not been validated in patients with chronic IF.^12^ In this current case, the LFTs of the patient were performed on average once every 6 months and they remained static from 2007 to 2011. However, his liver biopsies in 2009 and 2011 showed bridging fibrosis compared with the one performed in 2002, which showed cholestasis only. Although FIB‐4 score could not be used in this case, as patient was 21 years old, there is evidence that FIB‐4 scores do not correlate with the degree of histological fibrosis found in IFALD.^14^

FibroScan, also known as transient elastography, is a noninvasive, bed‐side test that measures the velocity of the sound wave as it goes through the liver and converts it into a liver stiffness measurement, which is expressed in kilopascals.^15^ It is used in patients at risk of advanced liver fibrosis to rule‐in advanced fibrosis or cirrhosis. However, existing literature shows the lack of correlation between FibroScan results and liver fibrosis in adult patients with chronic IF.^14^, ^16^ This may be due to liver stiffness being influenced by portal inflow and patients with chronic IF do not have normal mesenteric/portal hemodynamics.^17^ Bond and colleagues reported a case in which a patient with IF‐associated liver fibrosis was reduced from 7 days a week of SO ILE to 2 days a week of SO ILE before subsequent switch to SO‐MCT‐OO‐FO ILE.^18^ Despite the LFTs being unchanged, a FibroScan demonstrated worsening fibrosis, which prompted a liver biopsy. The liver biopsy contradicted the FibroScan finding by showing regression of fibrosis.^18^

A small pilot trial involving 20 adult patients with IF receiving long‐term PN, suggests that using the Liver Maximum Capacity test (LiMax), can help with early detection of hepatic dysfunction, thus highlighting those requiring subsequent liver biopsy.^19^ LiMax is a noninvasive breath test that assesses the liver function capacity rather than the degree of fibrosis, in which ^13^C‐labeled methacetin is injected intravenously and the liver subsequently converts it to paracetamol and ^13^CO_2_. This is then measured in the exhaled breath. The ^13^CO_2_/^12^CO_2_ ratios are continuously recorded via a proprietary device over a period of 60 min.^20^ LiMAx values of >315 mg/kg/h are considered normal.^21^ It is crucial to identify IFALD at an early stage when the patient can be referred for consideration of small bowel transplant prior to developing subsequent cirrhosis and its associated complications.^10^

Although IFALD is multifactorial and is not limited to PN,^22^ various aspects related to PN could contribute to IFALD, such as total number of days per week and total years receiving PN, total ILEs, glucose calorie intake, and type of ILE used.^22^, ^23^, ^24^ ILEs are required in PN as they provide essential fatty acids and allow for concentrated calorie provision to avoid high infusion loads of glucose, which has separate complications. The current patient was receiving SO ILE for 9 years, after which he developed bridging fibrosis, prior to switching to SO‐MCT‐OO‐FO ILE. However, it is important to note that the duration of time taken to develop liver fibrosis following the diagnosis of IF is variable in the literature,^24^, ^25^ as it can be influenced by other patient‐related factors such as sepsis, short bowel, and lack of enteral intake.^10^

A randomized open‐label trial assessed the impact of various ILE on liver biochemistry. Patients with chronic IF who were receiving long‐chain triglycerides (LCTs) were randomly assigned to receive MCT/LCT mixture, SO‐MCT‐OO‐FO ILE, or OO/SO ILE. At the end of 5 years, patients who were using SO‐MCT‐OO‐FO ILE had a significant reduction in bilirubin compared with their baseline levels at the start of the trial.^26^ Six years after the patient was switched to SO‐MCT‐OO‐FO ILE, his bridging fibrosis regressed and his LFTs improved. A possible explanation may be related to phytosterols, which is a component of SO that is structurally similar to cholesterol and is thought to contribute to hepatic steatosis when given intravenously because of its high bioavailability compared with when taken enterally.^27^ Through substitution of SO ILE with FO‐ILE, phytosterol content is reduced, whereas ω‐3 content is increased and ω‐6 content reduced, which can result in LFT improvement.^28^ Furthermore, a systematic review by Jones and colleagues in 2018, on the effects of various ILE, showed that the antioxidant status and LFTs improved with SO‐MCT‐OO‐FO ILE but not with an OO/SO ILE preparation.^29^

Although it is possible for IFALD to improve with reduction in parenteral ILE,^30^ the patient went from 7 days of SO ILE for 3 years to 3 days of SO ILE for a further 5 years prior to developing bridging fibrosis. However, in the case described by Bond and colleagues, the patient had been on seven nights of SO ILE under the care of the pediatric team for 18 years and initial liver biopsy showed prominent periventricular fibrosis. However, after transitioning to the adult team, his SO ILE was reduced to 2 nights per week and the lipid constituent remained at 100 g/day. Repeat biopsy 3 years after commencing SO‐MCT‐OO‐FO ILE and reducing ILE‐based PN to 1 night, his repeat biopsy showed histological improvement with minimal fibrosis. Bond's patient remained on 1 night of SO‐MCT‐OO‐FO ILE, but ILE dose was reduced to 50 g per day. Repeat histology 2 years later showed no fibrosis, but similar to the current patient, steatosis was observed.^18^ In both cases, the frequency of ILE nights was reduced and the number of glucose‐only nights increased. Although Bond did not report on the constituents of glucose‐only bags, the amount of glucose in the current patient's ILE‐free bags increased to accommodate for the reduction in ILE frequency (Table 1). Although our patient has always had a reference‐level hemoglobin A1c (<6.0%) and random glucose, it is possible that this change has contributed to hepatic steatosis.

Despite the patient receiving PN for a shorter period than Bond's patient, his histology showed worsening bile duct paucity between 2002 and 2011 and persistent duct paucity in 2015. The patient in Bond's report did not have bile duct paucity. In contrast, other studies have reported ductopenia in patients with IFALD. The first study to describe ductopenia in liver histology of patients with chronic IF was in 2011 by Nani and colleagues,^31^ the cohort included both adults and children without further analysis of prevalence of ductopenia in adults compared with children. Furthermore, the study reported ductopenia frequently develops in patients with low‐stage fibrosis, whereas the current patient had moderate bridging fibrosis instead. Finally, the researchers acknowledge that regression of fibrosis may be due to sampling variation, the patient's LFTs have remained stable and the liver transplant team that he is under do not require further liver biopsies.

## CONCLUSION

This case report further supports the currently, albeit sparse, adult long‐term PN literature showing that the use of SO ILE predisposes patients more to IFALD, but, more importantly, significant IFALD with fibrosis can be significantly improved with the use of newer ILE formulations. Furthermore, bridging fibrosis can potentially regress with conversion to third‐generation FO‐containing ILE formulations, such as SO‐MCT‐OO‐FO ILE. Although this patient continues to have some features of IFALD, the researchers propose that changes to the ILE to a third‐generation FO‐containing ILE has averted the need for small bowel or even multivisceral transplantation. Although a paucity of randomized controlled trials exist for the use of third‐generation ILEs, this case report supports the literature in showing the benefits of modern ILE prescribing.

## AUTHOR CONTRIBUTION

The concept and design of the study was jointly conceived by Sheldon C. Cooper, Richard Parker, and Rachael M. Brown. Shahd A. Mohamed, Sheldon C. Cooper, and Rachael M. Brown contributed to the acquisition of the data. The manuscript was drafted by Shahd A. Mohamed and Manoji Gamage. Sheldon C. Cooper and Shahd A. Mohamed contributed to the interpretation of the data. All authors critically revised the manuscript agree to be fully accountable for ensuring the integrity and accuracy of the work and have read and approved the final manuscript.

## CONFLICT OF INTERESTS STATEMENT

Dr S. Cooper had no conflict of interests at the time of this manuscript's submission. After submission, he received a paid honorarium from Takeda for chairing a meeting.
